# Retraction Note: Metagenome-assembled genomes infer potential microbial metabolism in alkaline sulphidic tailings

**DOI:** 10.1186/s40793-024-00582-5

**Published:** 2024-06-12

**Authors:** Wenjun Li, Xiaofang Li

**Affiliations:** 1 Hebei Key Laboratory of Soil Ecology, Key Laboratory for Agricultural Water Resource, Center for Agricultural Resources Research, Institute of Genetics and Developmental Biology, Chinese Academy of Sciences, Shijiazhuang, 050021 China; 2 University of Chinese Academy of Sciences, Beijing, 100049 China

**Retraction Note: Environmental Microbiome (2021) 16:9**

10.1186/s40793-021-00380-3

This article has been retracted by the Editor-in-Chief at the request of the authors because they did not have permission to publish the data presented. The scientific content has been removed for legal reasons. Both authors agree with this retraction.

